# miR-194-5p negatively regulates the proliferation and differentiation of rabbit skeletal muscle satellite cells

**DOI:** 10.1007/s11010-020-03918-0

**Published:** 2020-09-30

**Authors:** Yu Shi, Xudong Mao, Mingcheng Cai, Shenqiang Hu, Xiulan Lai, Shiyi Chen, Xianbo Jia, Jie Wang, Songjia Lai

**Affiliations:** 1 Farm Animal Genetic Resources Exploration and Innovation Key Laboratory of Sichuan Province, Sichuan Agricultural University, Chengdu, 611130 China; 2 Research Institute of Animal Husbandry of Ganzi Tibetan Autonomous Prefecture, Kangding, 626000 China; 3 College of Landscape Architecture and Life Science/Institute of Special Plants, Chongqing University of Arts and Sciences, Yongchuan, Chongqing, 402160 China

**Keywords:** Rabbit, Skeletal muscle satellite cell, miR-194-5p, Cell proliferation and differentiation

## Abstract

Skeletal muscle satellite cells (SMSCs), also known as a multipotential stem cell population, play a crucial role during muscle growth and regeneration. In recent years, numerous miRNAs have been associated with the proliferation and differentiation of SMSCs in a number of mammalian species; however, the regulatory mechanisms of miR-194-5p in rabbit SMSCs still remain scarce. In this study, miR-194-5p was first observed to be highly expressed in the rabbit leg muscle. Furthermore, both the mimics and inhibitor of miR-194-5p were used to explore its role in the proliferation and differentiation of rabbit SMSCs cultured in vitro. Results from both EdU and CCK8 assays showed that miR-194-5p inhibited the proliferation of SMSCs. Meanwhile, *Mef2c* was identified as a target gene of miR-194-5p based on the dual-luciferase reporter assay results. In addition, upregulation of miR-194-5p decreased the expression levels of *Mef2c* and *MyoG* during rabbit SMSCs differentiation on Days 3 and 7 of in vitro culture. Taken together, these data demonstrated that miR-194-5p negatively regulates the proliferation and differentiation of rabbit SMSCs by targeting *Mef2c*.

**Electronic supplementary material:**

The online version of this article (10.1007/s11010-020-03918-0) contains supplementary material, which is available to authorized users.

## Introduction

Skeletal muscle satellite cells (SMSCs), also known as a multipotential stem cell population, mainly exist on the surface of myofiber but beneath the basement membrane. Usually, adult SMSCs are quiescent, but they are prone to be activated by physical trauma or growth signals [1]. It has been widely accepted that SMSCs function as the myogenic precursors and give rise to myoblasts which eventually differentiate into multinucleated myotubes [2]. The myogenic basic-helix-loop-helix (bHLH) family of transcription factors, including *MyoD*, *MyoG*, *Myf5,* and *Mrf4,* is known as key regulators of myogenic differentiation of myoblasts. Silencing of *MyoG* resulted in a reduction of skeletal muscle tissue of mice by impeding the differentiation of myoblasts [3]. In addition, the members of the myocyte enhancer factor 2(Mef2) gene family, which recognize a conserved A/T-rich element of muscle-specific genes, are highly expressed in myoblasts. Previous studies have suggested that *Mef2c* and *MyoD* can regulate the differentiation of myoblast by co-activating their downstream target genes [4, 5].

MiRNAs, a class of non-coding RNAs, serve as posttranscriptional regulators of gene functions through base pairing with the seed sequence of 3′UTR region of genes, thereby interfering genes expression [6]. Over the last few decades, many studies have proved that miRNAs play important roles in a variety of cellular activities, including cell proliferation, differentiation, and apoptosis [7, 8]. Nowadays, lots of studies have indicated that miRNAs also play a role in myogenesis. For instance, miR-1 and miR-206 were upregulated during the differentiation of SMSCs, and upregulation of their expression promoted both the proliferation and differentiation of SMSCs [9]. miR-34a was shown to positively regulate smooth muscle cell differentiation by increasing the expression of *Mef2c*, *SRF* and *SirT1* [10]. And levels of miR-133a and miR-133b increased during the differentiation of human myoblasts [11]. Recently, increasing studies have focused on the regulation of satellite cells in domestic animals because of the major contribution of muscle to meat production. For example, the levels of Bta-miR-378 increased significantly during the differentiation of bovine skeletal muscle-derived satellite cells (bMDSC) and the over-expression of bta-miR-378 enhanced bMDSC differentiation [12]. Zhang et al. [13] found miR-143 promoted the differentiation of bovine SMSCs by targeting *IGFBP5*. miR-27b was identified as an activator of differentiation but an inhibitor of proliferation by targeting *MDFI* in porcine pig satellite cells [14]. Miao et al. [15] proved that miR-194 functioned as a suppressor of the proliferation and migration of osteosarcoma cells. Similarly, upregulation of miR-194 had decreased cell viability and increased apoptosis in prostatic cancer cells [16]. Apart from this, miR-194 family was found to associate with PI3K/Akt signaling pathway [17], which has been proved to be necessary for the proliferation and differentiation of myoblasts [18–20].

In this study, we aimed to investigate the expression of miR-194-5p in the leg muscle of rabbit and reveal the possible role of miR-194-5p in the proliferation and differentiation of rabbit SMSCs through gain and loss function of miR-194-5p. Our results showed that miR-194-5p negatively regulated the proliferation or differentiation by acting on *Mef2c*.

## Materials and methods

### Ethics statement

All animals used in this experiment were treated properly according to the “Guidelines for Experimental Animals” enacted by Ministry of Science and Technology (Beijing, China). This study was reviewed and approved by the Institutional Animal Care and Use Committee (IACUC) of Sichuan Agricultural University, under the permit No. DKY-B20141401.

### Sample collection and cell culture

Three New Zealand rabbits, aged 84 days, had free access to food and water. After being slaughtered, tissues (including heart, liver, spleen, lung, kidney, and muscle) were immediately collected and frozen in liquid nitrogen. SMSCs were isolated and purified as described in our laboratory [21]. SMSCs were cultured in growth media (GM) consisting of DMEM/HIGH GLUCOSE (DMEM) supplemented with 10% Fetal bovine serum (Gibco, Australia) and 2% penicillin streptomycin (Hyclone, USA). For differentiation induction, SMSCs were cultured in differentiation media (DM) consisting of DMEM with 2% horse serum (Beyotime, Shanghai, China) and 2% penicillin streptomycin (Hyclone, USA). All media had been changed every other day.

### Cell transfection

Transfection was conducted when SMSCs reached 70–80% confluence in 24 well or 96 cell. SMSCs were transfected with miR-194-5p mimics (50 nM; 5′–3′ Sense: UGUAACAGCAACUCCAUGUGGA; Antisense: CACAUGGAGUUGCUGUUACAUU), negative control (NC, 50 nM; Sense: UUCUCCGAACGUGCUACGUTT; Antisense: AGCUGACACGUUCGGAGAATT), inhibitor (100 nM; UCCACAUGGAGUUGCUGUUACA), and inhibitor negative control (inNC, 100 nM; CAGUACUUUUGUGUAGUACAA) using Lipofectamine 3000 (Invitrogen, USA), respectively. Cells were exposed to transfection regents for 24 h or 36 h and then incubated in GM or DM as needed.

### RT-qPCR

Total RNA was extracted from cells or tissues using RNAiso Reagent (Takara, Japan) according to the manufacturers’ protocol. The RNA concentration and purity were estimated using NanoDrop 2000 UV–Vis spectrophotometer (Thermo, Waltham, MA). The RNA quality was assessed using 1.5% agarose gel electrophoresis. The cDNA was synthesized using PrimeScript™ RT reagent Kit (Takara, Japan). RT-qPCR reaction was performed in triplicate using SYBR Premix EX Taq™II (Takara, Japan) and run on CFX96™ Real-Time PCR Detection System (Bio-Rad, USA). No-template controls and negative controls without cDNA template were also included in all qPCR runs. The relative expression levels of genes were normalized to the reference gene *GAPDH* using the 2^−△△Ct^ method [22].

The synthesis and quantification of miRNA were conducted using Mir-X™ miRNA qRT-PCR SYBR® Kit (Takara, Japan) according to the manufactures’ protocol. The relative expression of miRNA was normalized to reference gene *U6 snRNA* using the 2^−△△Ct^ method. All the corresponding primer sets are listed in Table S1.

### Cell proliferation assay

When cells seeded at 2 × 10^3^cells/well in 96-well plates reached about 70–80% density, transfection was conducted. 10 μL of Cell-Counting Kit-8 (CCK-8) reagent was added into wells for 2-h incubation at a 24-h interval. Each time the absorbance of cells was measured at a test wavelength of 450 nm using an automatic microplate reader.

In addition, EdU analysis was also conducted to explore the function of miR-194-5p in SMSCs proliferation. Cell preparation and transfection were also done as described previously. After transfection, cells were cultured in GM for 24 h. Then, SMSCs were incubated in medium containing 50 μM EdU for 2 h. Thereafter, cells were fixed, permeabilized, and stained by Hoechst 33,342 (1:1000) for 10 min at room temperature. In the end, images were obtained using a fluorescence microscope and the number of nuclei and EdU incorporation were analyzed using Image J software (National Institute of Health, Bethesda, MD). The percentage of EdU-positive cells was calculated by dividing the number of nuclei incorporating EdU by the number of total nuclei. 

### Luciferase reporter analysis

The target genes of miR-194-5p were predicted using online tools miRbase and TargetScan (https://www.mirbase.org/earch.shtml/https://www.targetscan.org/vert_71/). For luciferase assays, psiCHECK2-*Mef2c*-3′UTR (wild plasmid) and psiCHECK2-Mut-*Mef2c*-3′UTR (mutated plasmid) were constructed. Then wild plasmid or mutated plasmid were co-transfected with miR-194-5p mimics or NC into 293 T cells. After 36 h of incubation, cells were harvested and firefly luciferase (*luc2*) activity was measured and normalized to the *Renilla* luciferase (*hRluc-neo*) activity (*luc2/hRluc-neo*) using the Dual-Luciferase reporter assay system (TransGen Biotech, China).

### Target gene prediction and gene ontology enrichment analysis

Online software miRWalk (https://mirwalk.umm.uni-heidelberg.de/) was used to predict the target genes of miR-194-5p. As there were no complete data of rabbit in the current version of miRWalk, prediction was conducted based on the miRNA-mRNA interaction of *Mus musculus*. Then the target genes were analyzed through gene ontology (GO) term enrichment analysis and KEGG pathway analysis using DAVID 6.8 (https://david.ncifcrf.gov/).

### Statistical analysis

All data are expressed as mean ± SEM and were analyzed using GraphPad Prism 6.07 (GraphPad Software, San Diego, CA, USA). A *p*-value less than 0.05 indicates statistically significant difference.

## Results

### Bioinformatic analysis of the predicted target genes of miR-194-5p

**Fig 1:**
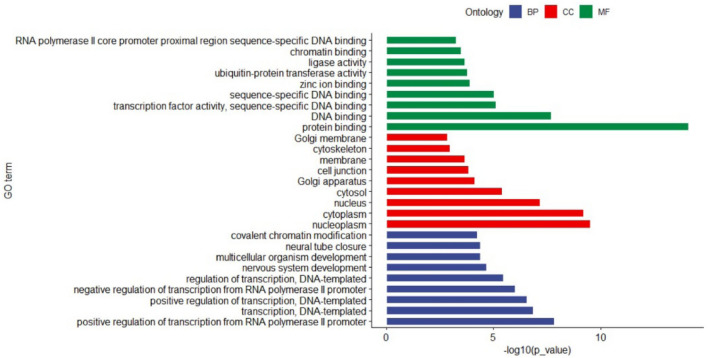
Go term analysis of miR-194-5p based on its predicted target genes that are involved in different signal pathways. *BP* biology progress, *CC* cellular component, *MF* molecular function

A total of 16,190 target genes of miR-194-5p were predicted using the miRWalk online software and then used for GO term analysis (Fig. 1). First, these targets were mainly enriched in 9 biology progresses, including RNA polymerase II core promoter proximal region sequence-specific DNA binding, chromatin binding, ligase activity, ubiguitin-protein transferase activity, zinc ion binding, sequence-specific DNA binding, transcription factor activity/sequence-specific DNA binding, DNA binding, and protein binding. Second, these target genes were related to several cellular components consisting of Golgi membrane, cytoskeleton, membrane, cell junction, Golgi apparatus, cytosol, nucleus, cytoplasm, and nucleoplasm. Third, the analysis of molecular functions of these target genes showed that they mainly play a role in covalent chromatin modification, neural tube closure, multicellular organism development, nervous system development, regulation of transcription/DNA-templated, negative regulation of transcription from RNA polymerase II promoter, positive regulation of transcription/DNA-templated, transcription/DNA-templated, and positive regulation of transcription from RNA polymerase II promoter. Furthermore, the results of KEGG pathway analysis showed that these targets were mainly enriched in several pathways, including ubiquitin-mediated proteolysis, signaling pathways regulating pluripotency of stem cells, TGF-beta signaling pathway, Wnt signaling pathway, MAPK signaling pathway, Axon guidance, protein processing in endoplasmic reticulum, ErbB signaling pathway, Rap 1 signaling pathway, and Hippo signaling pathway (Table 1).

**Table 1:** The KEGG pathways enriched with predicted targets of miRNA-194-5p

Pathways	Related genes	*p*-value
Ubiquitin-mediated proteolysis (mmu04120)	*SYVN1, UBE3A, BIRC6, FBXW7, UBE2D3, UBA2, UBR5, TRIM32, UBE2W, SMURF2, SMURF1, CUL4B, TRIP12*	47E-05
Signaling pathways regulating pluripotency of stem cells (mmu04550)	*FGFR1, IGF1R, RIF1, ID4, DUSP9, FZD5, BMPR1B, AKT2, FZD6*	0.0093
TGF-beta signaling pathway (mmu04350)	*LTBP1, ZFYVE9, TGIF1, SMURF2, ID4, SMURF1, BMPR1B*	0.0095
Wnt signaling pathway (mmu04310)	*CHD8, PRICKLE1, CAMK2G, PPP3R1, SOX17, FZD5, DAAM1, TCF7L2, FZD6*	0.0105
MAPK signaling pathway (mmu04010)	*MEF2C, FGFR1, PAK2, RASGRF2, TAOK1, PPP3R1, DUSP10, FGF10, RAP1B, DUSP9, AKT2, ATF2*	0.0195
Axon guidance (mmu04360)	*SEMA6A, NRP1, LIMK2, PAK2, EFNB2, NTNG1, PPP3R1, LRRC4C*	0.0203
Protein processing in endoplasmic reticulum (mmu04141)	*UBE2D3, SEC31B, SEC24B, TUSC3, SYVN1, TXNDC5, HSPA4L, NFE2L2, DNAJC1*	0.0273
ErbB signaling pathway (mmu04012)	*PAK2, ERBB4, CAMK2G, STAT5B, HBEGF, AKT2*	0.0381
Rap1 signaling pathway (mmu04015)	*FGFR1, IGF1R, PFN2, TLN2, CNR1, KRIT1, FGF10, RAP1B, PRKD3, AKT2*	0.0396
Hippo signaling pathway (mmu04390)	*CSNK1D, TEAD1, FZD5, BMPR1B, LATS1, PPP2R2C, TCF7L2, FZD6*	0.0427

### miR-194-5p expressed highly in rabbit leg muscle

**Fig 2:**
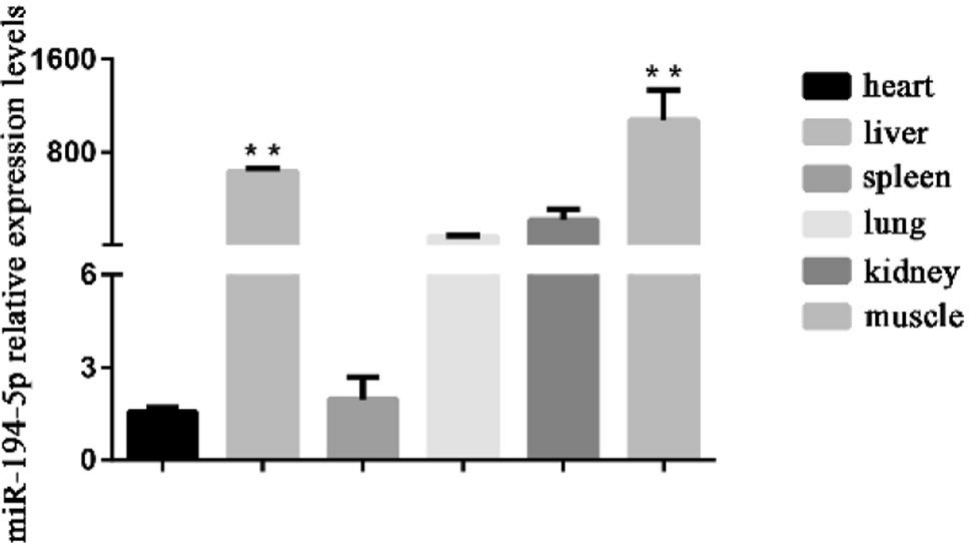
The expression levels of miR-194-5p in heart, liver, spleen, lung, kidney, and muscle of rabbits. Values are expressed as mean ± SEM of 3 individuals. *p < 0.05 or **p < 0.01 means the difference is significant

The expression levels of miR-194-5p in various tissues (heart, liver, spleen, lung, kidney, and muscle) were detected using RT-qPCR. The result showed that miR-194-5p expressed significantly higher in the liver and leg muscle of rabbits than that in other tissues (Fig. 2).

### miR-194-5p negatively regulates the proliferation of rabbit SMSCs

**Fig 3:**
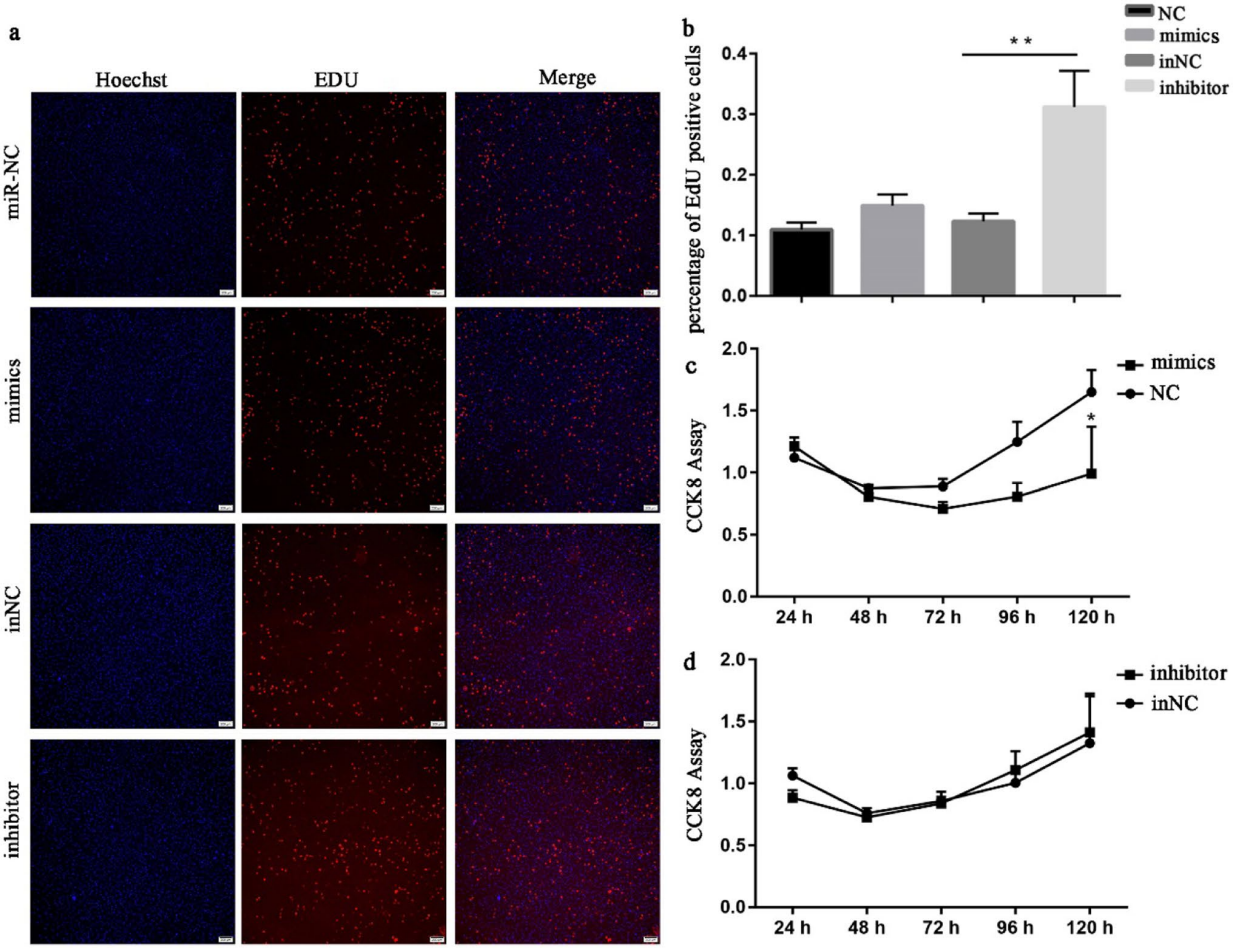
SMSCs were transfected with miR-194-5p mimics, negative control (NC), inhibitor negative control (inNC), and inhibitor for 48 h. The proliferation rate was represented by the percentage of EdU-positive cells **a-b** and CCK8 assay (**c-d**). The ratio of EdU-positive cells was calculated using the formula: (EdU-positive cells/ Hoechst stained cells) × 100% and the scale bar length was 200 μm. Values are presented as mean ± SEM of 3 pooled cells per group. *p < 0.05 or **p < 0.01 means the difference is significant

To investigate whether miR-194-5p plays a role in the proliferation of rabbit SMSCs, miR-194-5p mimics, inhibitor, and their negative controls were introduced into rabbit SMSCs, respectively. Next, both CCK-8 and EdU assays were used to detect the effects of miR-194-5p during the proliferation of rabbit SMSCs. The results of EdU assay showed that although there were no significant differences in proliferation of SMSCs between miR-194-5p mimics and NC groups, inhibition of miR-194-5p dramatically promoted the proliferation rate of SMSCs (Fig. 3a, b). On the other hand, the CCK-8 assay showed that miR-194-5p mimics significantly inhibited the proliferation of SMSCs at 120 h after transfection (Fig. 3c, d).

### *Mef2c* is identified as one of the target genes of miR-194-5p

**Fig 4:**
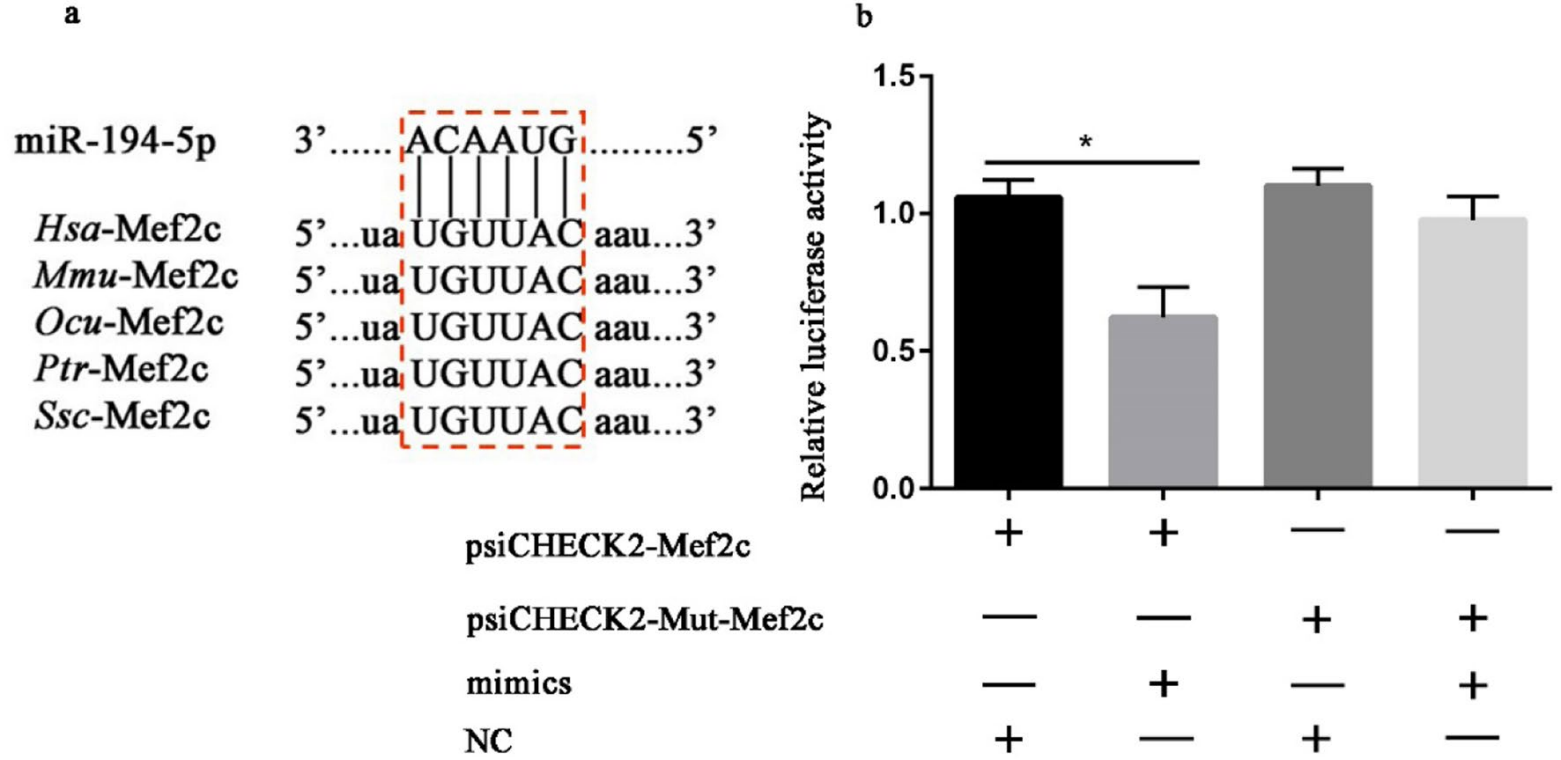
*Mef2c* is a target gene of miR-194-5p. The binding sites of miR-194-5p are completely conservative in Homo sapiens, Mus musculus, Oryctolagus cuniculus, Pan troglodytes, and Sus scrofa (**a**). psiCHECK2-Mef2c and psiCHECK2-Mut-Mef2c plasmids were co-transfected with miR-194-5p mimics or NC into 293 T cells and the activity of firefly luciferase normalized by Renilla luciferase activity (*luc2/hRluc-neo*) was measured for 6 replicates (**b**). *p < 0.05 or **p < 0.01 means the difference is significant

**Fig 5:**
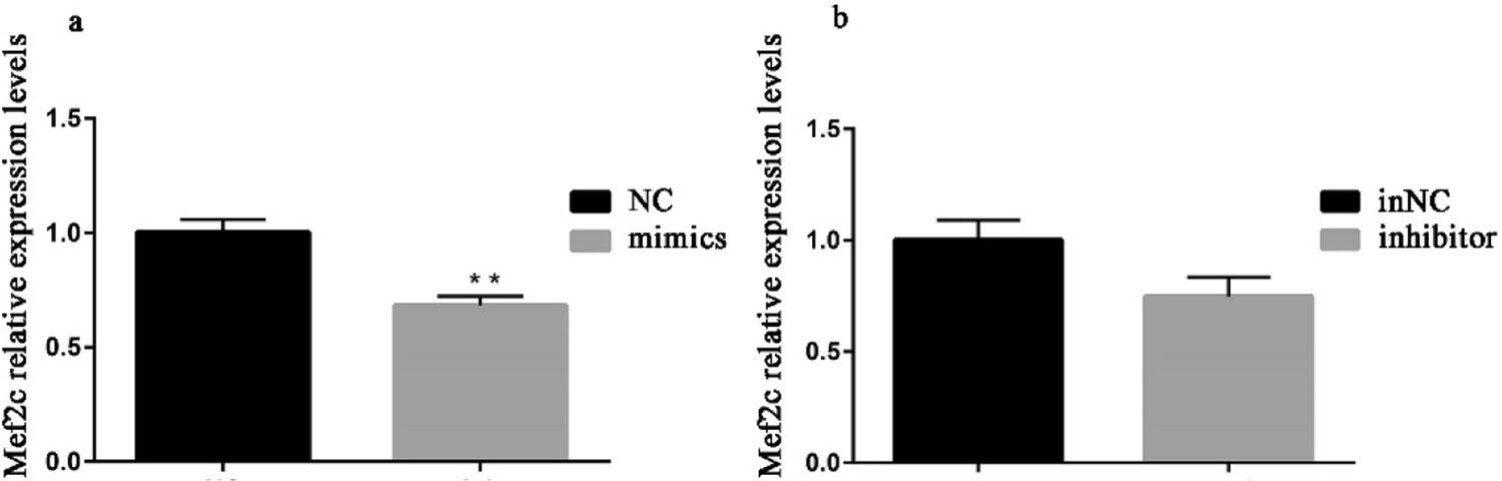
The effects of miR-194-5p on *Mef2c* expression levels. Values are expressed as mean ± SEM of 3 replicated cells per group; *p < 0.05 or **p < 0.01 means the difference is significant

According to the KEGG pathway analysis, *Mef2c* was one of the potential target genes of miR-194-5p and a key gene in MAPK signaling pathway (Table 1; Fig. 4a). To confirm this relationship, recombined plasmids (wild plasmid and mutated plasmid) were constructed. Then 293 T cells were co-transfected by wild or mutated plasmid with the mimics or NC of miR-194-5p and the relative activity of firefly luciferase was analyzed using dual-luciferase reporter system. The result showed that *luc2/hRluc-neo* relative activity was significantly reduced after co-transfection of miR-194-5p mimics and wild plasmid but remained unchanged in other treatments. (Fig. 4b). To further verify the relationship between *Mef2c* and miR-194-5p, the expression of *Mef2c* in rabbit SMSCs was measured after transfection of miR-194-5p mimics, NC, inhibitor, and inNC, and the results showed that over-expression of miR-194-5p significantly suppressed the *Mef2c* expression, but there was no significant difference between the treatments of miR-194-5p inhibitor and inNC (Fig. 5).

### miR-194-5p negatively regulated the differentiation of rabbit SMSCs

**Fig 6:**
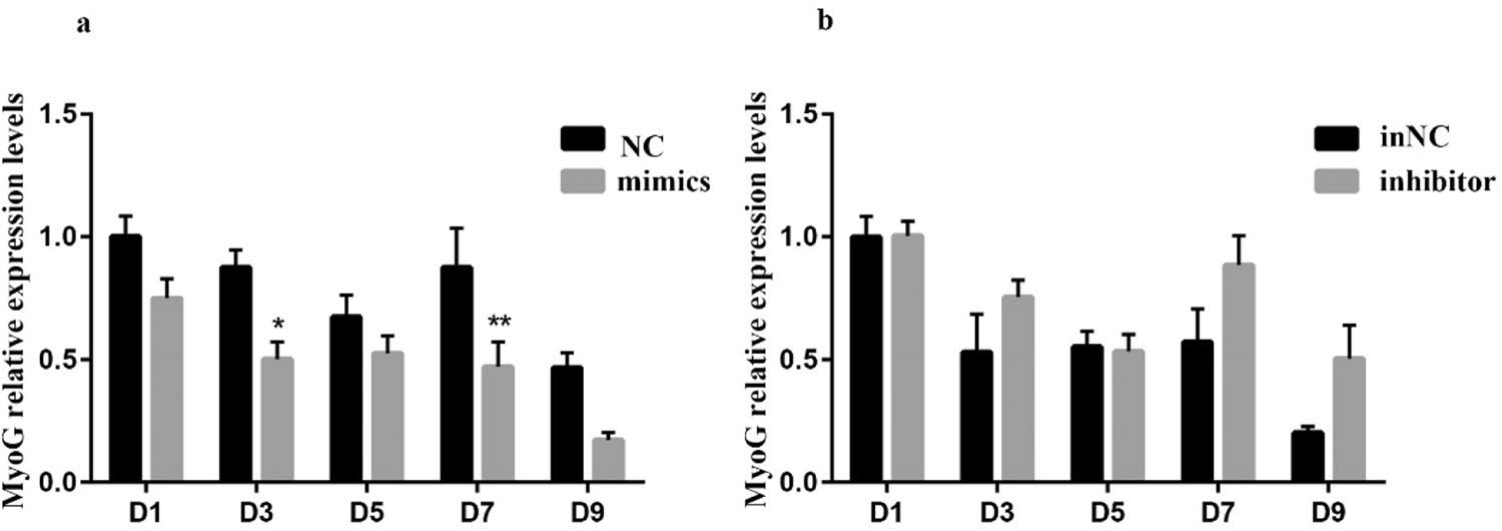
The effects of miR-194-5p on the expression levels of *MyoG* during myogenesis of SMSCs on D1, D3, D5, D7, and D9. Values are expressed as mean ± SEM of 3 replicated cells per group; *p < 0.05 or **p < 0.01 means the difference is significant

In order to define the role of miR-194-5p in the differentiation of rabbit SMSCs, differentiation of SMSCs were induced by replacing 10% FBS with 2% horse serum after 24-h transfection. During the differentiation of SMSCs, the expression levels of *MyoG* on Days 1, 3, 5, 7, and 9 were measured. The results showed that over-expression of miR-194-5p significantly suppressed the expression of *MyoG* on Days 3 and 7 during SMSCs differentiation. In contrast, inhibiting miR-194-5p expression had no significant effect on *MyoG* expression (Fig. 6).

## Discussion

In recent years, a growing number of studies have demonstrated that miRNAs play a vital role in myogenesis by targeting muscle-specific genes [23, 24]. For instance, Bjorkman et al. [25] showed that miR-206 and miR-1 could downregulate Srsf9 expression to promote the differentiation of C2C12 cells. Over-expression of miR-675-3p and miR-675-5p promoted the differentiation of mouse myoblasts [26]. Apart from myogenesis-associated genes and miRNAs, a lot of signaling pathways, including PI3K/AKT, are also involved in regulating myogenic differentiation [27]. To date, many researchers have identified miR-194-5p as a regulator of tumorigenesis. For example, over-expression of miR-194 significantly inhibited the proliferation and migration of gastric cancer cells [28], and the enhanced expression of miR-194-5p promoted Gallbladder cancer cell proliferation by targeting AKT2 gene [29]. These researches indicated that the effect of miR-194-5p on cell proliferation are cell specific. A previous study showed Zfb609 circular RNA negatively regulated mouse myoblast differentiation by sponging miR-194-5p [30], suggesting miR-194-5p may play a positive role in the myogenesis of mouse myoblasts. However, it is unclear whether miR-194-5p play a role in the proliferation and differentiation of rabbit SMSCs.

Bioinformatics analysis of miR-194-5p displayed the potential functions of its target genes and several enriched signal pathways, like Wnt and MAPK signaling pathways, playing a role in myogenesis. Wnt/PCP pathway has been shown to play a role in the symmetric expansion of satellite cells [31]. Besides, activation of Wnt-β-catenin signaling induced follistatin and myogenin to promote myoblasts differentiation [32]. It has been proved that p38α promotes myoblasts differentiation by preventing cell proliferation [33]. Moreover, the activation of p38-MAPK pathway could promote the fusion of myoblasts to myotubes [34]. In the study, the expression of miR-194-5p was detected in various tissues of rabbits for the first time. RT-qPCR analysis showed miR-194-5p expressed the highest in leg muscle of rabbits, indicating that miR-194-5p might play a role in rabbit skeletal muscle development. It has been known that activated satellite cell pool served as a cell reservoir for the maintenance, hypertrophy, and repair of adult muscle [2]. The classic view proposed by Moss and Leblond [35] pointed that on account of the asymmetrical cell division, the population of SMSCs is heterogeneous containing myoblast precursor cells and quiescent satellite cells, which made SMSCs self-renew possible. Thus, it seems proliferation is the precondition of the formation of myoblast precursor cells. In our study, the EdU and CCK-8 assays showed that inhibiting miR-194-5p expression significantly promoted the proliferation rate of SMSCs, whereas over-expression of miR-194-5p suppressed the proliferation of SMSCs at 120 h after transfection, suggesting that miR-194-5p negatively regulated the proliferation of SMSCs. Until now, a lot of myogenesis-associated genes have been well defined, among which MEF-2 was widely expressed in muscle cells (cardiac, skeletal, and smooth muscle cells) [36]. It has also been reported that MEF-2 acts as a downstream target of the bHLH proteins (*MyoD*, *MyoG*, *Myf5*, etc.), and co-activation of MEF-2 and these bHLH proteins can activate the majority of muscle-specific genes [37]. Furthermore, Mef2c has been identified to repress gene expression by interacting with histone deacetylases and responding to various signaling pathways to activate gene expression after calcium influx, activation of calcineurin, and activation of MAPK signaling pathway [38–40]. In this study, *Mef2c* was first identified as one of the target genes of miR-194-5p using dual-luciferase assay. Upregulation of miR-194-5p in rabbit SMSCs significantly decreased the expression of *Mef2c*. Besides, the expression levels of *MyoG* on Day 3 and Day 7 during myogenesis was reduced by over-expression of miR-194-5p. Studies have proposed that *Mef2c* worked as a signal mediator of MAPK signaling pathway in cells [41, 42]. So, the results suggested that miR-194-5p inhibited the differentiation of rabbit SMSCs via inhibition of MAPK signaling pathway by acting on *Mef2c*.

In summary, miR-194-5p was revealed to be highly expressed in the leg muscle of rabbits. At the cellular level, it negatively regulated the proliferation of rabbit SMSCs. Moreover, the over-expression of miR-194-5p decreased the expression of *MyoG*, suggesting that miR-194-5p inhibited the differentiation of rabbit SMSCs by inactivating the MAPK signaling pathway through targeting *Mef2c*.

## Electronic supplementary material

Below is the link to the electronic supplementary material.

Supplementary file1 (docx 15 kb)
