# Supplementary material for: miR-194-5p negatively regulates the proliferation and differentiation of rabbit skeletal muscle satellite cells
Source: Mol Cell Biochem. 2020 Sep 30;476(1):425–33. doi: 10.1007/s11010-020-03918-0 (PMC7867548; doi:10.1007/s11010-020-03918-0)
Supplement: Supplementary file 1 — Supplementary file1 (docx 15 kb) [file 11010_2020_3918_MOESM1_ESM.docx]

**Table S1** Information of primers used for qRT-PCR

| Gene names | Primer sequence(5'→3') | Fragment Size | Tm/℃ |
| --- | --- | --- | --- |
| *MyoG* | F:GAGACATCTCCCTACTTCTACCA  R: GCTCAGTCCGCTCGTAGCC | 106 | 59 |
| *Mef2c* | F: AAGATAAACAGAAATGCTGAG  R: CAATGAGTGCCATACGC | 378 | 57 |
| *GAPDH* | F: CTTCGGCATTGTGGAGGG  R: GGAGGCAGGGATGATGTTCT | 130 | 57 |
